# Supplementary material for: Are carbon emissions trading and green financial instruments synergistic? -Comprehensive quantitative research based on content analysis
Source: PLoS One. 2024 Mar 7;19(3):e0298601. doi: 10.1371/journal.pone.0298601 (PMC10919601; doi:10.1371/journal.pone.0298601)
Supplement: S1 Data — (ZIP) [file pone.0298601.s001.zip › Data/地级市Z_F工作报告.zip!/║┌┴·╜¡/╥┴┤║╩╨/└┤╫╘╣1⁄2╓┌║┼í╛┬φ┐╦╩2╛▌═°í┐_2019.docx]

各位代表：

现在，我代表市人民政府向大会报告工作，请予审议，并请市政协委员和列席会议的同志提出意见。

一、2018年工作回顾

2018年是伊春转型发展历程中极不平凡的一年，是林区改革深化进程中具有里程碑意义的一年。肩负新使命，面对新挑战，在市委的领导下，我们坚持以习近平新时代中国特色社会主义思想为指导，深入贯彻落实党的十九大精神，贯彻落实习近平总书记在深入推进东北振兴座谈会上重要讲话，以及对我省重要讲话和指示精神，按照省市委全会部署要求，狠抓工作推进，强化工作落实，较好地完成了市十四届人大二次会议确定的各项目标任务，全市经济社会向着高质量发展目标迈出了坚实步伐。

——经济运行持续稳中向好。坚持以转方式调结构为重点，充分发挥重大投资、重点项目和龙头企业牵动作用，全力稳增长扩投资促消费，主要经济指标赶上全省平均水平。预计全年地区生产总值实现275亿元、同比增长6%，规上工业增加值实现27亿元、增长4%，固定资产投资完成92亿元、增长8%，社会消费品零售总额实现141亿元、增长8%，进出口总额实现5.1亿元、增长10%，公共财政预算收入完成17.9亿元、增长10.5%，城镇常住居民人均可支配收入达到25290元、增长7%，农村常住居民人均可支配收入达到14730元、增长7%。

——三大攻坚战扎实推进。在脱贫攻坚上，着力补齐基础设施和产业扶贫短板，仅市级财政就投入资金1.2亿元，用于完善贫困村供水、道路、广场、卫生所等基础设施，发展扶贫产业项目71个。全市19个省级贫困村全部出列，嘉荫县和南岔区整体脱贫，贫困发生率降至0.48%。在污染防治上，深入开展环境综合治理，加强废弃菌袋、畜禽养殖污染防治，饮用水水源水质达标率保持100%，淘汰中心城区燃煤小锅炉92台，秸秆露天禁烧取得阶段性成果，全市空气质量优良天数达98.6%，综合指数位居全省前列。在防范化解重大风险上，建立政府债务动态监管平台，严控增量，化解存量，债务风险总体可控，有效整治了金融乱象。

——重点产业发展质效齐升。围绕打造“两座金山银山”、做好“三篇大文章”，突出一产抓融合、二产抓提升、三产抓拓展，推动生态重点产业加快发展。森林生态旅游业持续升温。巩固提升了森林观光、避暑度假、温泉滑雪等传统优势产品，创新发展了民宿研学、汽车营地、康体养生等新业态。成功举办东北亚森林博览会、中国金融40人伊春论坛、伊春国际森林马拉松等品牌会展赛事。“森林深处”“冰雪森林”冬夏主题产品纳入全省中部核心产品体系。荣获“中国最具潜力避暑旅游城市”“十佳魅力中国城”等殊荣。全年游客数量和旅游收入均实现25%以上增长。森林食品业规模扩大。各类种养基地达261处，蓝莓等小浆果种植面积达7万亩，食用菌达6.2亿袋，绿色有机作物达295万亩。森林猪、森林鸡、雪貂饲养量分别达53万头、45万只和18万只。汇源伊佳食品有限公司推出蓝莓果汁、蓝靛果果汁、木耳茸饮，以及花青素、红松籽坚果油、速冻山野菜、蓝莓果干、果酱、果饼等系列产品。忠芝、新昊、北货郎等一批森林食品企业市场占有率不断扩大。“伊森林”“小兴安岭大森林”品牌日益叫响。北药业初见端倪。南岔、汤旺河、红星、铁力局4个“森林百草园”和金南公路北药产业带规模逐步扩大，葵花、格润、金北药等规上药企实现产值4.3亿元，同比增长32%，企业加工能力持续提升。木业加工业创新发展。光明集团智能家具生产线建成投产，永达、美江等木艺企业开发新品，龙头牵动作用逐步增强，全市家具和木艺行业产值分别同比增长4%和8.3%。绿色矿业效益提升。西钢集团成功破产重整，实现产值102亿元、利税9.6亿元。鹿鸣钼矿产销两旺，实现产值21亿元、利税11亿元。华能热电发电和供热量双增，实现产值12亿元、利税2.3亿元。翠峦、铁力等重点园区累计入驻企业101户，承载集聚能力不断提升。

——生态建设基础更加坚实。深入践行习近平生态文明思想，全面加强资源保护管理，国家重点生态功能区作用日益凸显。认真实施天保二期工程，坚持依法治林，查处涉林案件199起，收回林地24.5公顷。小兴安岭—三江平原山水林田湖草生态保护修复项目，经财政、规划、国土、环保部门争取，获得国家批复，涉及伊春项目投资18亿元，为加快建设绿色美丽伊春提供了项目资金支持。森防现代化建设不断加强，购置森防车辆203台、风力灭火机1600套，实现连续15年未发生重大森林火灾。科学经营森林，培育后备资源14万亩，抚育中幼林280万亩。

——各项改革取得积极成效。国有林区改革实现历史性突破，伊春森工集团挂牌成立，林业局企业办社会职能稳妥有序剥离，延续60年的政企合一体制走向终结。重点领域改革不断深化，“放管服”改革力度加大，政府机构改革如期推进，行政区划调整方案上报国家待批，以供热集团为代表的国企和事业单位改革进展顺利。对外交流稳步拓展。援疆工作有序开展。

——城乡建设步伐加快。全力补短板、强弱项、扩投资，一批打基础利长远的重大基础设施项目顺利实施。“大通道”建设加快推进，铁力至伊春高速铁路项目完成前期，伊汤公路建成通车，汤嘉公路获国家批复，溪水经营所至林都机场等3条旅游公路主体完工，林都机场改扩建工程进展顺利，市客运总站主体建成。保障性安居工程全部开工，改造棚户区6111户、老旧小区30个，完成既有建筑节能改造16万平方米。“三供三治”建设全面提速，西山水库净水厂建成投用，改造主城区供热一级网1.4万米，天然气入户2000户。乌伊岭、西林等7个垃圾和污水处理厂建成运营。更新电动公交车50台，增开了翠峦公交线路。乡村振兴战略全面启动，全市规上农产品加工企业达66户，99个村屯实现安全饮水，50个村屯动力电工程建成投用，新建农村公路86公里。扎实推进农村人居环境整治三年行动，村容村貌明显改观。

——林区群众获得感切实增强。公共财政支出85%投向民生领域，年初承诺的12件民生实事进展顺利。持续推进就业增收，城镇登记失业率低于省控指标，森工企业职工人均月增资400元。社会保障能力逐步提高，城乡低保和特困人员供养政策全面落实，102个病种纳入医保范围，企业基本养老金实现连续14年上调。社会事业加快发展，学前三年毛入园率高于省均4个百分点，在全省率先整体通过县域义务教育基本均衡国家验收，高考实现13连增，职业学院学生公寓、医学楼等4项工程全部完工。第五医院和中医院改扩建顺利完成，市中心血站、残疾人康复中心主体竣工，妇幼保健院建成投用。全力应对洪涝灾害，积极推进灾后重建。扫黑除恶专项斗争取得阶段性成果，安全生产形势持续好转，社会大局保持和谐稳定。

——行政效能不断提高。全面转作风、优环境、争一流，政府执行力和公信力有效提升。着力推进政务服务事项“一窗受理、一网通办、一站式服务”，市本级服务事项全部纳入政务大厅，平均办结时间缩短54.8%，“网上办”“最多跑一次”事项占比分列全省第2和第4位。建成全省第一个地市政府采购网上交易平台，大额采购形成制度。工程招投标进一步规范。人大代表意见建议和政协委员提案回复率100%。

各位代表，回顾过去一年的工作，我们遇到的困难比预想的要大，经受的考验比预料的要多。之所以能够顶住压力、应对挑战、突破困难，取得来之不易的成绩，主要得益于市委的坚强领导、统筹推进，得益于市人大和市政协的依法监督、民主监督、大力支持，得益于全市干部群众的担当尽责、苦干实干。在此，我代表市人民政府，向全市人民以及所有支持、参与伊春建设的各界人士，表示衷心的感谢和崇高的敬意！

与此同时，我们也清醒地认识到，政府工作还存在诸多不足、面临不少挑战。主要是：发展不充分、经济总量小、发展速度慢、发展质量不高、内生动力不足；企业数量少、规模小、财税贡献率低；思想解放程度不够，产业市场化程度偏低，稳增长调结构的压力还很大；深化改革难点多、任务重，需要破解的矛盾还很多；林区群众的生产生活条件有待进一步改善，脱贫攻坚、就业增收、乡村振兴的任务还很艰巨；审批流程不优、行政效率不高、干部作风不实等问题还不同程度地存在，重塑营商环境仍然任重道远。对此，我们将坚持问题导向，采取有力措施，以不畏艰难的勇气、坚韧不拔的意志，尽最大努力加以解决！

二、2019年工作安排

今年是新中国成立70周年，是全面建成小康社会关键之年。按照市委部署，政府工作的总体要求是：以习近平新时代中国特色社会主义思想为指导，全面贯彻落实党的十九大和中央经济工作会议精神，聚焦贯彻落实习近平总书记在深入推进东北振兴座谈会上的重要讲话和考察我省的重要指示精神，统筹推进“五位一体”总体布局，协调推进“四个全面”战略布局，坚持稳中求进工作总基调，坚持新发展理念，坚持推进高质量发展，坚持以供给侧结构性改革为主线，落实“六稳”要求，把握“八字方针”，坚决打好三大攻坚战，统筹推进稳增长、促改革、调结构、惠民生、防风险，以“让老林区焕发青春活力”为统领，围绕打造“两座金山银山”，解放思想，扬长避短、扬长克短、扬长补短，做好“三篇大文章”，重塑投资营商新环境、重聚产业发展新动能、重构协调发展新格局、提升绿色发展优势、提升改革开放水平、提升民生保障能力，全力推动经济平稳健康发展和社会大局稳定，开创伊春全面振兴全方位振兴新局面，以优异成绩迎接新中国成立70周年。

主要预期目标是：地区生产总值增长5%以上，固定资产投资增长8%，规模以上工业增加值增长3%，公共财政预算收入增长8%，社会消费品零售总额增长6.5%，进出口总额增长2%，城乡居民收入增长与经济增长同步。

要实现上述总体目标，做好今年经济工作，我们必须准确把握发展形势，积极抢抓战略机遇，主动应对风险挑战，以更加坚定的信心和决心，改革开放再出发，打好发展组合拳，走好绿色转型路。在实际工作中：要深刻把握全面振兴全方位振兴的新要求。把振兴发展的着力点放在全面贯彻新发展理念上，放在结合实际、与时俱进谋划工作上，解放思想、锐意进取，瞄准方向、保持定力，以问题为导向，全面深化重点领域改革，不断深化区域开放合作，加快培育壮大新动能，持续巩固提升绿色发展优势，切实保障和改善好民生，努力走出一条质量更高、效益更好、结构更优、优势充分释放的振兴发展新路。要深刻把握深化供给侧结构性改革的新内涵。坚决贯彻落实“巩固、增强、提升、畅通”八字方针，更多采用改革办法，更多应用市场化、法治化方式，巩固“三去一降一补”成果，切实减轻企业负担；增强市场主体活力，加快民营经济发展；提升优势产业链水平，培育壮大重点产业；畅通国民经济循环，提高金融服务实体经济能力，努力把八字方针贯穿于推动高质量发展的全过程。要深刻把握加快绿色转型发展面临的新机遇。紧紧抓住国家推进东北振兴重大战略机遇期、窗口期，全面落实国家经济结构优化升级重大部署，在放大生态资源优势上持续发力，在解决产业结构偏重、民营经济偏弱、创新人才偏少“三偏”问题以及体制机制、经济结构、开放合作、思想观念“四个短板”上力求实效。对接国家战略，以目标为导向，加快推动一二三产同向发力，不断壮大实体经济，突出抓投资、抓项目、抓招商，努力将生态优势转化为经济优势、产业优势、竞争优势，努力为全市人民创造更多财富，创造更好生活。

（一）坚决打好三大攻坚战，夯实全面建成小康社会基础。紧紧抓住各项攻坚任务，针对突出问题，打好重点战役，确保与全省、全国同步全面建成小康社会。

打好脱贫攻坚战。落实落靠市、县（区）、乡各级政府扶贫主体责任，坚持将脱贫攻坚作为第一民生工程，突出“精准”原则，务实推进。强化产业扶贫，重点抓好铁力市、嘉荫县、南岔区1000栋木耳大棚建设，提高产业项目覆盖率。加强“两不愁三保障”“三通三有”基础设施建设和政策落实，确保剩余455户、710人全部脱贫。切实巩固脱贫成果，提高群众满意度，坚决做到脱真贫、真脱贫、不返贫。打好防范化解重大风险攻坚战。持续整治金融乱象，健全完善地方政府债务风险应急处置和联合监测防控机制，严格实施防范债务风险责任考核，规范举债行为，多渠道处理存量债务，切实防范化解债务风险。打好污染防治攻坚战。继续强化秸秆露天焚烧监管，积极推进秸秆离田还田，秸秆综合利用率达到75%。严禁建成区新建35蒸吨以下燃煤锅炉，加快淘汰建成区外10蒸吨以下燃煤锅炉。加强重点流域水污染防治。加强饮用水水源地保护区规范化建设。加强水土流失综合治理。抓好废弃菌包综合利用项目建设，严控重点景区、公路两侧、自然保护区及水源地范围内大地摆栽。强化畜禽养殖污染物排放治理，全市规模养殖场污染处理设施配套率达到80%以上。持续打好蓝天、碧水、净土保卫战，让伊春的天更蓝、山更绿、水更清。

（二）全面加强生态建设，加快创建国家生态文明先行示范区。立足国家重点生态功能区定位，实施最严格的保护制度，统筹抓好山水林田湖草系统治理，提升生态系统的质量和稳定性。

切实加强生态资源保护。深入推进“严管林”，依法严厉打击盗伐林木、蚕食林地、毁林开荒、掠青采集等各类破坏林地、林木和湿地资源的违法行为，从严查处乱捕滥猎和非法经营野生动物违法活动，有序推进退耕还林、还草、还湿。深入推进自然保护区“一区一法”建设，扎实开展“绿盾”专项行动。积极推动汤旺河石林、五营红松原始林申报世界自然遗产。加强森防基础设施和专业队伍建设，确保不发生重特大森林火灾。全面启动小兴安岭——三江平原山水林田湖草生态保护修复工程，统筹实施南北山林缘交错带生态修复、南北山植被恢复和保护、伊翠铁路生态修复、伊春河水环境治理、伊春区秋沙鸭栖息地修复治理等工程项目。实施好伊春河北岸地质环境修复项目。编制矿山地质环境恢复和综合治理规划。推动自然资源资产离任审计。做好天保工程三期数据测算和政策争取。加快构建现代森林经营体系。科学培育后备森林资源，重点抓好铁力、美溪、友好和红星4个森林经营试点局建设，提升27个国营苗圃标准化建设水平，苗木产能达到1亿株。强化造林质量管理，全年培育后备资源8万亩，抚育中幼林270万亩。推动国家战略储备林基地建设。探索发展碳汇经济，稳步开展碳增汇试点工作。严格落实生态保护主体责任。全面落实中央环保督察“回头看”整改任务。抓好生态保护红线划定工作。落实落靠“河长制”，严守水资源“三条红线”。

（三）持之以恒转方式调结构，培育壮大经济增长新动能。坚持以深化供给侧结构性改革为主线，围绕“粮头食尾”“农头工尾”，大力培育新的增长点，推动实体经济高质量发展，加快构建现代林业经济新体系。

一产突出提高品质。加快推动绿色有机农业发展。突出优质、特色、绿色有机调整优化农业种植结构，水稻、玉米、杂粮杂豆、设施蔬菜面积分别达到73万亩、62万亩、9万亩和1万亩。全面落实粮食安全市长责任制。推进高标准农田建设，实施农业“三减”示范130万亩，新增绿色有机食品认证水稻基地21万亩，力争“三品一标”作物达到300万亩。加强农田水利建设，实施呼兰河以及铁力、嘉荫等中小河流治理工程，推进铁力小白水库建设前期工作，新增高效节水灌溉5万亩。推进特色渔业发展，冷水鱼养殖面积达到8900亩。推进生态畜牧业发展，力促铁力金新农100万头、嘉荫林大牧业30万头生猪项目建成投产，适度发展肉牛、奶牛、羊和禽类，加强非洲猪瘟等疫病疫情防控。着力提高农业生产机械化水平，积极培育各类新型经营主体，力争新增5个农机专业合作社、5个市级农民示范合作社、20个家庭农场。加强与农业院校、科研院所对接，为农业插上科技翅膀。二产突出增加效益。通过盘活存量、扩大增量，推动实体经济发展，增强工业经济牵动力。加大重点企业支持力度，推动建龙西钢、鹿鸣钼矿、华能热电、北方水泥、光明家具等骨干企业做大做强。强化为骨干企业服务意识，支持企业技术改造、提高产品质量、扩大产能、提高产量。强化生产要素保障，积极落实减税降费政策，更多争取电力直接交易政策，降低企业成本。稳定规上工业运行，完善规上企业培育储备机制，扶持汇源伊佳、林宝生物等一批“临规”企业升规入统，对效益不好的规上企业，要因企施策，分类指导，减少“退规”。加快园区建设，铁力园区重点打造绿色产业发展平台，翠峦园区打造森林食品生态产业园，西林园区打造清洁生产全产业链冶金产业园，南岔园区打造北药、生物医药产业园，力争各类园区新入驻企业15户以上。园区要重点做好清理整顿，僵尸企业要尽快出清，有效资产要尽快盘活，闲置土地要依法收回再招商。三产突出创新拓展。着力推动产品创新、技术创新、业态创新和商业模式创新，促进传统服务业提档升级，现代服务业加速发展。有序发展旅游地产、康养地产，着力提升美溪廻龙湾、伊春岐黄、新青华仕康旅居等康养基地的影响力。加快发展会议会展经济，举办好魅力中国城旅游产业博览会、中国金融40人伊春论坛以及第四届全国黑木耳产业创新发展大会。加快发展电商物流产业，推进“1林GO”等各类电商平台建设，提高电商产业园、汇源物流园企业入园率。加快促进文化、体育、旅游融合发展，办好国际森林马拉松等赛事。加快发展现代金融服务业，优化金融生态环境，加强金融市场监管，积极引进银行、保险、证券、担保以及小额贷款公司等各类金融企业，创新金融产品和金融服务，全市存贷比要上升5%。推动一二三产融合发展。围绕粮食、林果、林木、矿产资源进行精深加工，提供游客“必购必带”的旅游产品。积极发展沟谷经济，加快推进金山屯九峰山养心谷、上甘岭溪水等特色山庄提档升级，着力完善铁力香草河、五营“自在香里”等山水田园综合体功能，打造休闲农业和乡村旅游精品工程。着力打造产业融合载体，加快推动铁力现代农业产业园、友好国家农村一二三产融合发展先导区建设，促进田园采摘、休闲观光、研学体验等产业深度融合，推动一产接二连三，延伸产业链，提升价值链。

（四）深挖优势资源潜力，加快推动重点产业提档升级。围绕改造升级“老字号”、深度开发“原字号”、培育壮大“新字号”，放大伊春整体生态优良、资源丰富、林木产业基础雄厚等优势，做好“绿水青山”“冰天雪地”文章，加快形成多点支撑、多业并举、多元发展的产业格局。

推动全域旅游示范区加快建设。承办好第二届全省旅发大会，努力打造全省样板和模式。坚持“重点打造、整体提升、规划引领、分步实施”的原则，重点打造汤旺河石林、五营和上甘岭溪水森林公园、嘉荫恐龙馆和茅兰沟等核心景区，重点推出伊春—汤旺河—嘉荫北部森林生态观光精品线路，重点展示森林观光、自驾休闲、民宿体验、地质探秘等特色产品，实现游客“必到必游”。整体提升避暑度假、康体养生、林海雾淞、滑雪狩猎等优势产品供给能力，丰富拓展西岭旅游度假区、桃山森林冰雪玉温泉、铁力日月峡等景区，完善提升红星大平台杜鹃花海、铁力依吉密河漂流、朗乡万松岩五花山、金山屯冰湖雪村等景区，推动带岭岭秀、新青中小学研学基地建设，力促四季均衡发展。加快智慧旅游平台建设，建立旅游数据中心，完善升级“一部手机游伊春”APP功能，实现旅游景区4G全覆盖。加强导游等从业人员培训培养，提升接待服务能力。规划引领产业发展，编制“多规合一”全域旅游发展规划，抓好与产业发展、生态保护等规划的衔接融合，严肃规划执行，避免低水平重复建设。分步实施各项重点工作，今年上半年实施好伊春龙建旅游客运公司组建，购置旅游客车30辆；汤旺河、五营汽车营地，汤旺河欧陆风情、上甘岭溪水等特色民宿，汤旺河森林生态主题科普基地建设。下半年完成凤凰水岸美食风情一条街，以及景区游客服务中心、停车场、旅游厕所等设施建设。集智集力打造一台高水准、高质量的开幕式演出，全面彰显伊春地域文化特色。利用三年时间实施哈伊高速小兴安岭段、伊嘉公路两侧绿化美化治理工程，打造“北国百里风景观光廊道”。强化整体营销，深化与各大旅行社、旅游联盟合作，力争深度融入全省营销线路格局。深化国有景区体制机制改革，引进战略投资者和专业管理团队，推进4A级以上景区改制，加快产业市场化发展步伐。力争全年入境游客数量和旅游收入均实现两位数增长。推动森林食品业提质增效。加快特色种养基地建设，重点扩大小浆果、小坚果、食用菌、山野菜等标准化基地规模，力争全市蓝莓、蓝靛果种植面积达到7.5万亩，黑木耳达到7亿袋、棚室达到7000栋。严格执行森林猪饲养标准，力争森林猪、森林鸡、雪貂分别达到55万头、50万只和20万只。强化精深加工，进一步提高蓝莓等浆果果汁、果酒、花青素，红松籽等坚果果油，黑木耳、液态菌等产品供给质量和品牌建设能力。整合矿泉水品牌，重点推动高锶、泓源等企业开工生产。推动北药业产业化发展。按照GAP标准抓好单品种标准化基地建设，重点扩大刺五加、苍术、返魂草、平贝等骨干品种规模。支持葵花、喜人、格润、金北药等龙头企业加快技术改造和产品研发，提高道地药材就地加工增值能力。推动铁力四宝林蛙油、汤旺河今生康健饮片、南岔格润药业植物提取等项目达产达效。探讨与安国药材大市场深度合作，尽早进入全国药材销售网络，全面提升“林都北药”影响力。推动木业加工业创新提升。支持光明等龙头企业精准对接市场需求，扩大环保、家装、木制建材等产品订单化生产，提升市场竞争力。支持美江、永达、侨艺、凯瑞林等木艺企业提高创意设计水平，推出更多“北沉香”、木艺根雕、核桃花瓶、木艺版画等产品，叫响“中国木艺之乡”。推动绿色矿业增产扩能。坚持“开发一点，保护一面”，着力提升矿产品持续供给能力。重点支持建龙西钢围绕产能配套、环保和安全进行改造，实现400万吨产量和420万吨产能。鹿鸣钼矿要提高采矿和加工能力，原矿处理量达到1500万吨。支持华能热电新上供热锅炉，既扩大供热面积，又形成热源“双保障”。加强同海派玉雕协会合作，切实提升“北红玛瑙”“桃山玉”产品档次和价值。持续跟进省地质勘查三年专项项目，加快西林外围铅锌矿等探矿项目进度，力争在矿泉水、地热、玉石等新兴矿种勘查方面实现新突破。

（五）突出抓好项目建设，切实增强经济发展后劲。坚持把项目建设作为推动转型发展的重要抓手，深入实施项目建设三年攻坚战，谋划储备一批、开工建设一批、达产达效一批、对接争取一批，以项目稳增长、稳就业、稳投资。全年安排亿元以上重点项目70个，总投资272亿元，年度计划投资87亿元。

推进产业项目建设。建立项目建设专班，落实落靠项目包抓责任制，确保盯得住、落得下、建得快。重点推进建龙西钢130万吨焦化、100万吨双高线，以及铁力葵花药业中药前处理提取、伊春药业提取车间、南岔格润药业综合生产车间、友好丰园森林食品改扩建等18个新开工项目加快建设，推进铁力局恒硕桦树汁产业园、嘉荫恐龙地质公园2号馆等12个续建项目投产投用。继续充实完善市、县（区）两级重点产业项目库，设立项目谋划专项资金。重点围绕绿色生态产业发展，扎实谋划一批投资大、效益好、对地方经济拉动强的高质量项目。全年谋划超5000万元项目100个以上，超亿元项目50个以上。推进基础设施项目建设。突出抓好大通道建设，开工建设时速250公里双线铁力至伊春高速铁路，建设汤旺河至嘉荫一级公路，完成林都机场改扩建工程并投入使用，推动五营区址至森林公园、带岭朝阳至铁力丰林林场、南岔区址至仙翁山景区3条旅游公路建成通车。加大招商引资力度。适时出台招商引资优惠政策，建立“专班+园区”“目标+考核”招商机制，大力开展专业招商、以商招商、委托招商，各县区（局公司）至少引进亿元以上项目1个。全力抓好对上争取，加强与国家、省相关部门沟通对接，力争更多项目挤进国家和省计划“盘子”，最大限度地获得政策资金支持。鼓励铁力、嘉荫立足资源、产业优势，差异化发展，切实提高抓项目、抓招商、抓财源的能力和水平，培育壮大县域经济。

（六）全面深化改革开放，进一步激发内生动力。坚持问题导向，充分发挥改革对实现高质量发展的推动作用，做实改革举措、释放改革活力、提高改革效能，推动林区大改革、大转型、大发展。

深化林业综合改革。推动森工集团既坚持公益属性，发挥好承接天保工程管护经营森林资源的主体作用，又加快建立现代企业制度，提升市场经营能力，带动林业局公司、林场所发展林下经济，促进林业职工创业增收。以“南四局”公司为试点，适时启动17个林业局自然资源资产盘点，为下步整合资源、形成股份合作打下基础。推动伊林集团、伊旅集团加快发展。推进政府机构改革，重点抓好人员转隶、职能划定等工作，确保如期完成改革任务。稳步推进行政区划调整。深化国有企业改革。加快事业单位改革。推进重点领域改革。继续落实“三去一降一补”改革任务，努力降低企业制度性交易成本和生产要素成本。加快推进农村集体产权制度改革，筹备建设产权交易中心。完成全市公安体制改革和森林公安改革任务。重塑营商新环境。进一步深化“放管服”改革，完善权力清单、责任清单和负面清单制度。继续精减审批、再造流程，推进“证照分离”“多证合一”，将企业开办时间压缩到8个工作日以内。深化招投标领域改革，简化手续，并联审批，缩短时间。持续推进“互联网+政务服务”，大力推行一网通办、在线办结等服务方式，做到“网上办”不见面，“一次办”少跑腿，“我帮办”有温度。整治窗口服务顽疾，着力打造高效透明的政务环境、公平正义的法治环境、诚信守约的人文环境、绿水青山的生态环境以及风清气正的政治生态环境。提升对外开放水平。深化对俄合作，积极推进嘉荫口岸复关。扩大对外交流，加强与德国、罗马尼亚和加拿大等国友好城市，在文体、旅游、医养等领域的交流合作。推动与广东茂名对口合作，力争在食品加工、医药生产、避暑康养度假地产等领域，落地一批标志性跨区域合作项目。开展好援疆工作，全面落实对口援建框架协议。

（七）深入推进创新创业，不断激发市场活力和社会创造力。突出发挥创新创业驱动作用，持续提升创新创业能力水平，催生更多市场主体，留住更多宝贵人才，培育更多新的动能。

提高科技创新能力。深入实施创新驱动发展战略，安排市级以上重点科技项目20项。落实“科技型企业三年行动计划”，推动林科院、农研中心等科研院所与企业加强合作，开展食用菌、小浆果等优良品种培育，以及蓝莓防寒、森林食品机械采收等科研攻关。完善科研人员激励机制，提高科技转化率，促进成果产业化。扎实推进国家知识产权试点城市建设。支持民营经济发展。贯彻落实国家和省对民营经济的支持政策，落实《市委、市政府支持民营经济发展实施意见（18条）》，建立中小企业行业协会。继续搭建政银企对接平台。支持金融企业创新产品。政府拿出引领资金，引进融资性担保公司，为小微企业提供融资担保、过桥贷款担保，着力破解企业融资难、融资贵问题。引导企业规范合法经营，依法加大清欠追缴力度，实行“黑名单”制，增强金融企业信贷投放信心。注重培养高素质企业家队伍，尊重企业家首创精神，畅通企业反映困难和诉求渠道，构建亲清新型政商关系，真正让民营企业放心、放手、放开发展。强化人才引进和培养。实施“头雁”行动，把引进人才、留住人才放在更加突出位置，加大平台、资金、政策、环境支持力度，促进各类人才创新创业。加强“院士工作站”“人才驿站”建设。完善国家级和省级小微企业创业创新基地孵化功能。推动职业学院、林校、技师学院提高职业技能教育水平，大力培养一批旅游、森林食品企业需要的本土实用技术人才。

（八）坚持城乡协同发展，加快建设美丽伊春。突出基础设施领域投资，进一步完善城市功能、提升城市品位，促进乡村（林场所）振兴，让城乡更美丽、更舒适、更宜居。

推进基础设施建设。实施廻龙湾大街等道路改造升级工程。推动市客运总站以及金山屯、汤旺河、五营客运站建成投用。完成既有建筑节能改造40万平方米。对市区部分二级供热管网进行改造，实施旧小区给水分户改造5000户，新增天然气用户800户。建设沿河景观公园、铁路公园、湿地景观，实施重点区域及主要道路节点绿化美化工程，让林都伊春更整洁、更靓丽。深入实施乡村振兴战略。坚持农业农村优先发展，对标全面建成小康社会必须完成的硬任务，推动农业增效、农民增收、农村发展。编制实施全市乡村（林场所）振兴战略规划。投入7000万元饮水安全工程资金和2000万元乡村振兴专项资金，推动基础设施、公共服务向乡村（林场所）延伸。改造建设通村公路22条85公里。加快实施林场所搬迁，推动深山区场所向中心林场集聚。全面开展人居环境整治三年行动，加快农村污水设施建设，建立城乡一体化垃圾收运体系，新建改建厕所1030个，推动乡村、林场所环境面貌大改观、大变样。提高城市管理水平。推进城市总体规划、专项规划修编，做好城市空间立体设计。加快智慧城市建设，拓展伊春时空信息云平台应用，发展智能交通。抓好地下管网建设，强化供水系统在线监测。持续推进“六项整治”，加强小区物业、集贸市场管理。规范出租车市场秩序，严厉打击“黑车”运营。建立健全长效机制，巩固创卫、创文成果。

（九）着力补齐民生领域短板，让林区群众共享振兴发展成果。坚持以人民为中心的发展思想，积极回应人民热切期盼，持续加大投入，解决百姓最关心最直接最现实的利益问题，让林区群众享有更多获得感、幸福感、安全感。

健全社会保障体系。突出保基本兜底线，推动退休人员基本养老金以及低保、特困保障标准提标工作，适度提高医保核销比例。推进医保信息联网。提升养老机构服务质量，加强养老机构安全管理。做好退役军人接收安置和优抚工作。全面治理拖欠农民工工资问题，切实保障劳动者报酬权益。加快发展社会事业。坚持把稳就业摆在突出位置，拓展高校毕业生、农民工、退役军人、林业富余职工等群体就业渠道，全年实现城镇新增就业1.9万人，城镇登记失业率控制在4.5%以内。坚持教育优先发展。继续推进标准化幼儿园建设，支持公办幼儿园、扶持民办幼儿园发展。推动义务教育由基本均衡向优质均衡迈进。推动省市示范高中建设，要实现高考本科入段率14连增。加快市中小学生综合实践学校建设，提升研学教育能力。充分发挥社区大学职能，推进社区教育。深入开展“森林之声”系列文化活动，完善公共文化服务体系。推进“健康伊春”建设。大力开展全民健身运动，新建市滑冰馆、休闲健身园，将公共体育场馆全方位向社会开放。稳步推进公立医院综合改革。启动市120急救中心建设。强化与省内知名医院合作，全面提升医疗服务水平。强化社会综合治理。深化“平安伊春”建设，继续推进“雪亮工程”，深入开展扫黑除恶专项斗争，巩固扩大治安防控成果。抓好安全生产，加强重点行业领域安全监管和专项整治，坚决遏制重特大事故。进一步畅通信访渠道，及时回应化解合法诉求，解决不动产登记历史遗留问题。强化社区治理服务。

三、切实加强政府自身建设

打铁还需自身硬。新时代、新目标、新任务更需具有新气象、体现新担当、展示新作为。我们必须要牢固树立“四个意识”，坚定“四个自信”，坚决做到“两个维护”，不折不扣落实中央和省市委各项决策部署，切实提升执政能力，全面提高行政效能，努力建设让人民满意的法治政府、务实政府、廉洁政府和服务型政府。

坚持依法行政。深入推进“法治伊春”建设，提高运用法治思维和法治方式开展工作、解决问题、推动发展的能力。进一步健全完善重大行政决策机制和程序，规范招投标、政府采购、公共资源交易等平台运行管理。加大政务公开力度，着力推进政务决策、执行、管理、服务和结果“五公开”，以公开促落实、促规范、促服务。加强“诚信伊春”建设，把诚信施政作为重要准则，挂牌督办一批政府失信违诺、新官不理旧账典型案件，不断增强政府执行力和公信力。自觉接受人大法律监督、政协民主监督以及社会各界监督，让公共权力运行更加阳光透明、规范高效。

持续推进作风转变。深入开展“解放思想推动高质量发展大讨论”活动，在解放思想中发现不足、找准差距、解决问题。加大政府系统作风整顿力度。增强狠抓落实本领，对重大决策、重点项目、重要工作要挂图作战、压茬推进。自觉践行为民宗旨，各级政府工作人员要主动到基层去、到生产现场去，特别是要到脱贫攻坚一线去，真诚倾听群众呼声，真实了解群众意愿，真心解决群众疾苦。提升调研能力，力戒形式主义和官僚主义。强化政府系统凭专业说话、按程序办事，用专业思维、专业素养、专业方法推动工作。勇于担当责任、敢于啃“硬骨头”，坚决做到矛盾面前不回避、困难面前不退缩。建立容错纠错机制，推动广大干部放开手脚履职尽责、干事创业，推动各项工作落到实处、见到实效。

强化廉洁从政。时刻将纪律和规矩挺在前面，坚决落实中央八项规定，持之以恒推进正风肃纪。着力完善重点部门和关键岗位廉政风险防控措施，强化对国有资产、工程项目、惠民资金等重点领域的审计监督。树立过紧日子思想，继续压缩行政成本，大力度节约集约财政资金和政府资源，确保“三公经费”零增长。严格履行主体责任和“一岗双责”，持续加强政府系统党风廉政建设，严厉惩治侵害群众利益的不正之风和腐败问题，以干部清正、政府清廉、政治清明的实际成效赢得人民群众的信任和支持。

各位代表，船到中流浪更急，人到半山路更陡。伊春已经进入转型发展的关键期、攻坚期，已经站在全面振兴全方位振兴的崭新起点上。新的征程引领我们勇往直前，神圣使命激励我们无畏艰险，人民期盼鼓舞我们不懈奋斗。让我们紧密团结在以习近平同志为核心的党中央周围，在市委的坚强领导下，解放思想、坚定信心，锐意进取、埋头苦干，加快推动伊春绿色转型发展，让老林区早日焕发青春活力！以优异成绩迎接新中国成立70周年！
